# Malaria and Hypertension: What Is the Direction of Association?

**DOI:** 10.1093/function/zqae037

**Published:** 2024-08-30

**Authors:** Aparna Tiwari, Auley De, Abhinav Sinha

**Affiliations:** ICMR—National Institute of Malaria Research, New Delhi 110077, India; ICMR—National Institute of Malaria Research, New Delhi 110077, India; ICMR—National Institute of Malaria Research, New Delhi 110077, India; Academy of Scientific and Innovative Research, Ghaziabad 201002, India

## A Perspective on “Hypertension Increases Susceptibility to Experimental Malaria in Mice”

The recent paper by Kandalgaonkar et al.^1^ was read with high interest as this is the only publication that raised the possibilities of an association between malaria and hypertension with an inverse direction of association. The authors tried to establish, although in mice, that a hypertensive state increases the “susceptibility” to malaria. This paper is of interest to the scientists working in this area because all the previously published literature hypothesized that exposure to malaria may predispose an individual to an elevated risk of hypertension through direct (inflammatory) and indirect (genetic) mechanisms.^2^ The authors^1^ performed comprehensive analyses on the pathophysiological dynamics of *Plasmodium* infection in hypertensive mice, showing, for the first time, that malaria pathology may be prolonged in hypertension. These findings are intriguing for both researchers and physicians as they may be helpful in explaining the co-occurrence of malaria and hypertension in certain areas of the globe. However, Kandalgaonkar et al.^1^ leave us with certain concerns that need attention of researchers working in the area so that the validity of the conclusions drawn by the authors may be further ascertained.

Models of hypertension and malaria: Human hypertension is a complex and multifactorial disease for which a perfect animal model is still a distant dream. Although a spontaneously hypertensive rat model is preferred, mouse models are not a good choice.^3^,^4^ This is particularly true for the genetically modified BPH/2 mice used by Kandalgaonkar et al.,^1^ which is a good model for neurogenic hypertension but not for vascular hypertension.^5^,^6^ Therefore, choice of animal model for hypertension is critical for external validity of the experimental results using animal models.^3^ The same is true for using the rodent model, *P. yoelli*, for malaria. *Plasmodium berghei* and *P. yoelli* have a strong predilection for invading reticulocytes, which might mimic human-infecting parasites, *P. vivax* and *P. ovale*, but not for *P. falciparum*. In addition, the blood stage duration of *P. yoelli* is ∼18 h in contrast to ∼48 h for *P. falciparum* and *P. vivax*.^7^ Therefore, *P. yoelli* might not be a good choice for using them to represent the erythrocytic stages of *Plasmodium*, particularly where reticulocyte dynamics are altered as reported by Kandalgaonkar et al.^1^ This limitation could be partly circumvented by using blood from hypertensive and non-hypertensive individuals for establishing *ex vivo*/*in vitro* cultures of *P. falciparum* parasites and not only comparing the parasitemia progression but also assessing the stage-specific progression of the parasite growth and development across multiple erythrocytic cycles.

Red blood cell (RBC) anomalies in hypertension and *Plasmodium* infection: RBCs have impaired membrane deformability (reduced fluidity/increased viscosity) in hypertension, which results in a stiffer membrane.^8^ Although the complete biomechanics of *Plasmodium* invasion in red cells is not fully decoded, it has been shown that raised RBC membrane tension deters parasite invasion^9^,^10^ with a bidirectional threshold tension range beyond which invasion is extremely unlikely.^11^ In addition, increasing RBC membrane fragility has been shown to significantly lower the intraerythrocytic parasite growth.^12^,^13^ Hypertensive state is also associated with increased RBC osmotic fragility (OF) in humans,^8^ but Kandalgaonkar et al.^1^ showed decreased RBC OF in hypertensive mice with high membrane rigidity. These findings favor a reduced surface/volume of RBCs or a laxed RBC membrane akin to a relatively dehydrated RBC,^14^ and the same is supported by the blood picture (microcytosis) of hypertensive mice. Both high (HbE) and low (HbAS, thalassemia, and HbAC) OF states deter parasite invasion and growth in RBCs and thus offer protection/resistance to malaria.^13^,^14^ Therefore, high “susceptibility” claimed by the authors may not be explained by altered RBC dynamics in hypertensive mice. Further, reticulocytes are considered to be osmotically more stable as compared to mature erythrocytes,^15^ and the same might be true in hypertension. Considering compensatory reticulocytosis, the OF curve might have been biased toward shifting to the left as reported by Kandalgaonkar et al.^1^ However, compensatory reticulocytosis might lead to some increase in parasitemia, which can compensate for the lower parasite invasion and growth due to the above-mentioned RBC anomalies. Targeted *in vitro* studies may be done to confirm the role of hypertension-induced RBC anomalies, particularly across different RBC OF gradients, to assess the effects on parasite invasion, growth, and egress as suggested by published literature.^9^,^14^,^16^,^17^

Susceptibility and sustained parasitemia: Because the term “malaria” is a clinical entity that is determined by demonstration of intraerythrocytic asexual form of the parasite in peripheral blood coupled with a febrile state that is unexplained otherwise, the term “susceptibility” to malaria implies that RBCs are more prone/favorable to parasite invasion/growth/development/replication and/or the infected person is more prone to develop fever at a lower parasitemia. Since RBC membranes are already fragile in hypertension and this fragility is further increased by *Plasmodium* infection, it is expected that the egress of the merozoites is prematurely precipitated by the fragility and therefore the duration of intraerythrocytic cycle is shortened and accompanied by slow intraerythrocytic growth, which is expected to lead to a lower number of merozoites per schizont and therefore lower parasitemia. This is contrary to what authors concluded.^1^ Further, transient sustained parasitemia, as shown by the authors, may not be termed increased susceptibility to malaria but rather be reflected as altered pathology. It would have been more pertinent if the authors had tried to dissect the intraerythrocytic stage-specific progression of the parasites in both the hypertensive and the non-hypertensive groups.

Thus, it is not expected that the RBC anomalies in hypertensive state per se will promote parasite invasion and/or sustained parasite infection. However, a hyper- or pro-inflammatory state, compromised immunity, and/or compensatory reticulocytosis could prematurely precipitate the symptoms of malaria and sustain infections with parasites that prefer reticulocytes for invasion and therefore make an individual more “susceptible” to malaria. However, such pathologies are ubiquitous and may not be restricted to hypertension. Although Kandalgaonkar et al.^1^ is a seminal step forward in assessing the association of malaria and hypertension from a novel perspective and has opened a fascinating area for further research, we believe that the claim that hypertension increases malaria “susceptibility” looks a bit premature at the moment considering the evidence cited by the authors. To further test the hypothesis, we suggest using blood from hypertensive patients and RBCs with anomalies found in hypertension for *P. falciparum* culture to assess the role of these variables independently. However, with malaria and hypertension both being clinical entities with complex pathophysiology, the ultimate way to test this hypothesis would be to conduct a cohort study in areas where both these diseases are prevalent such as in sub-Saharan Africa and certain regions in India.
